# Reticulate Evolution in the Western Mediterranean Mountain Ranges: The Case of the *Leucanthemopsis* Polyploid Complex

**DOI:** 10.3389/fpls.2022.842842

**Published:** 2022-06-17

**Authors:** Salvatore Tomasello, Christoph Oberprieler

**Affiliations:** ^1^Department of Systematics, Biodiversity and Evolution of Plants (With Herbarium), University of Göttingen, Göttingen, Germany; ^2^Evolutionary and Systematic Botany Group, Institute of Plant Sciences, University of Regensburg, Regensburg, Germany

**Keywords:** Anthemideae, AllCoPol, BEAST2, *Leucanthemopsis*, polyploidy, reticulate evolution, western Mediterranean

## Abstract

Polyploidization is one of the most common speciation mechanisms in plants. This is particularly relevant in high mountain environments and/or in areas heavily affected by climatic oscillations. Although the role of polyploidy and the temporal and geographical frameworks of polyploidization have been intensively investigated in the alpine regions of the temperate and arctic biomes, fewer studies are available with a specific focus on the Mediterranean region. *Leucanthemopsis* (Asteraceae) consists of six to ten species with several infraspecific entities, mainly distributed in the western Mediterranean Basin. It is a polyploid complex including montane, subalpine, and strictly alpine lineages, which are locally distributed in different mountain ranges of Western Europe and North Africa. We used a mixed approach including Sanger sequencing and (Roche-454) high throughput sequencing of amplicons to gather information from single-copy nuclear markers and plastid regions. Nuclear regions were carefully tested for recombinants/PCR artifacts and for paralogy. Coalescent-based methods were used to infer the number of polyploidization events and the age of formation of polyploid lineages, and to reconstruct the reticulate evolution of the genus. Whereas the polyploids within the widespread *Leucanthemopsis alpina* are autopolyploids, the situation is more complex among the taxa endemic to the western Mediterranean. While the hexaploid, *L. longipectinata*, confined to the northern Moroccan mountain ranges (north–west Africa), is an autopolyploid, the Iberian polyploids are clearly of allopolyploid origins. At least two different polyploidization events gave rise to *L. spathulifolia* and to all other tetraploid Iberian taxa, respectively. The formation of the Iberian allopolyploids took place in the early Pleistocene and was probably caused by latitudinal and elevational range shifts that brought into contact previously isolated *Leucanthemopsis* lineages. Our study thus highlights the importance of the Pleistocene climatic oscillations and connected polyploidization events for the high plant diversity in the Mediterranean Basin.

## Introduction

Polyploidy is the presence of three or more complete chromosome sets in an organism. The frequency of whole-genome duplications (WGDs) is heterogenous throughout the tree of life, and it is particularly prominent in plants. The ancestor of all angiosperms was polyploid (Jiao et al., 2011) and further rounds of WGD occurred in different angiosperm lineages during the Cretaceous–Paleogene boundary (Vanneste et al., 2014). Approximately 15% of speciation events in angiosperms involved an increase in the number of complete chromosome sets and their further diversification resulted in 35% of extant angiosperms being polyploid (Wood et al., 2009). The study of polyploid complexes and polyploid formation is therefore fundamental to improving our understanding of plant evolution.

Polyploid species are not evenly distributed on the Earth, with polyploid frequencies increasing toward high latitudes (Rice et al., 2019). Additionally, polyploidy is relatively frequent in plants of the Mediterranean Basin, where it has been a driving force for diversification in several genera, e.g., *Campanula* L. (Crowl et al., 2017; Liveri et al., 2020), *Centaurea* L. (Garcia-Jacas et al., 2009; Moreyra et al., 2021), *Centaurium* Hill., (Mansion et al., 2005), *Leucanthemum* Mill., (Oberprieler et al., 2014), and *Veronica* L. (López-González et al., 2021). About 30% of plant species in the whole Mediterranean biome are polyploids (Rice et al., 2019), with similar estimates given for the European Mediterranean Basin (36%; Marques et al., 2018). In the particular case of the Iberian Peninsula, numbers are even higher and approximately 50% of all plant species, including angiosperms, are polyploids (Marques et al., 2018).

Polyploidy is often linked to hybridization (i.e., allopolyploidy). In contrast to autopolyploids, which contain elevated numbers of sub-genomes from an individual or from different individuals of the same species, allopolyploids derive from the merging of two or more chromosome sets from different species (Ramsey and Schemske, 1998; Van De Peer et al., 2017). Of the two processes, allopolyploidy has always been considered to be more common (Stebbins, 1950; Grant, 1981, Coyne and Orr, 2004; but see Ramsey and Schemske, 1998, 2002; Soltis et al., 2007). The reticulate character of allopolyploid evolution makes inference of phylogenies in those groups particularly difficult. Moreover, the increased number of chromosome sets exacerbates problems connected with gene paralogy, and the effect of stochastic factors intrinsic to evolution, such as incomplete lineage sorting (ILS), becomes more dramatic as a consequence of the increased effective population size. Disentangling between hybridization and ILS in (allo)polyploids is not a simple task (e.g., Avise, 1994; Rosewich and Kistler, 2000; Linder et al., 2003), and only a few methods have been proposed in the last decade that are capable of reconstructing reticulate evolution in polyploid complexes (Oxelman et al., 2017; Rothfels, 2021).

Among those methods, AlloppNET and AlloppMUL are capable of modeling polyploid evolution and producing phylogenetic networks and multi-labeled (MUL-)species trees in the presence of both ILS and hybridization (Jones et al., 2013; Jones, 2017b). Unfortunately, the model is computationally demanding and has been designed to infer phylogenies in relatively small datasets, including diploids and a single tetraploid species. Recently, Yan et al. (2021) extended a maximum parsimony method for inferring phylogenetic networks in the presence of ILS and hybridization (Yu et al., 2013) to complexes including polyploids. However, the method needs prior knowledge on the mode of polyploid formation (i.e., the number of reticulations must be specified in advance). Moreover, alleles of polyploid species/samples are not sorted into sub-genomes; a piece of information that might be needed for further and more specific analyses (e.g., species delimitation of polyploids, age estimation, and post-formation genome evolution).

Other methods utilize iterative approaches to sort alleles of polyploids into sub-genomes and then infer MUL-species trees and networks. Some of these methods use information from the plastid genome to determine which of the homeologs found in nuclear genes are from the maternal progenitor by looking at (in)congruence between the plastid phylogeny and the nuclear gene trees (Bertrand et al., 2015; Šlenker et al., 2021). Problems arise when dealing with ploidy levels higher than 4*x* (hexaploids may potentially comprise three different sub-genomes) or in cases when the maternal progenitor of a polyploid is extinct or not sampled, as often reported in polyploid complexes (Oberprieler et al., 2014; Karbstein et al., 2022). The program, ALLCOPOL (Lautenschlager et al., 2020) uses the number of deep coalescences to sort alleles into parental sub-genomes and does not need prior information on polyploid formations and/or maternal progenitors. It is based on the combinatoric approach described in Oberprieler et al. (2017), but the implementation of heuristics and machine-learning algorithms makes it applicable to larger datasets including high polyploids.

**FIGURE 1:**
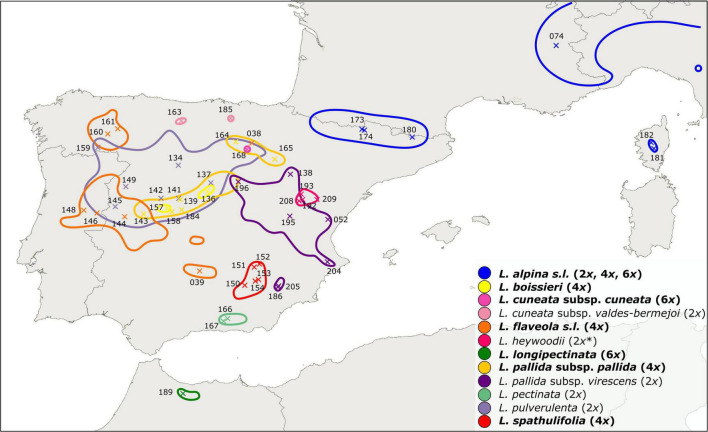
Distribution map of *Leucanthemopsis* taxa in the western Mediterranean as inferred from literature and herbarium records (Supplementary Figure 2; Supplementary Data Sheet 4). Taxa in bold are polyploids or include polyploid populations and the corresponding ploidy levels follow the names. * Diploid ploidy of *L. heywoodii* was inferred by our flow-cytometric measurements, whereas Pedrol (2019) reported this taxon to be tetraploid. ×, geographic position of the populations included in the present study with corresponding IDs (“LPS” in Supplementary Data Sheet 1).

**FIGURE 2:**
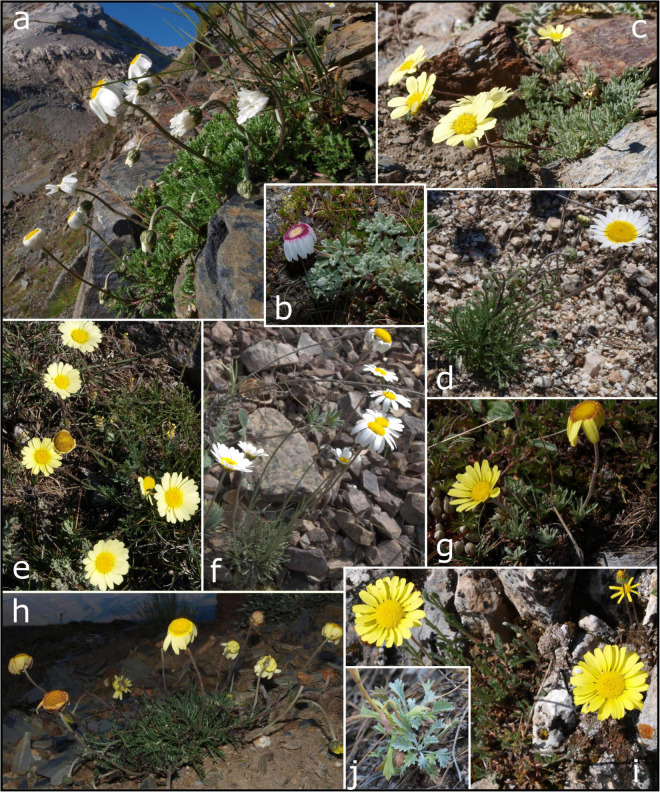
Morphological diversity in *Leucanthemopsis*: **(a)** *L. alpina* subsp. *pyrenaica* (Port de Boucharo, Hautes-Pyrénées, France); **(b)** *L. alpina* subsp. *tomentosa* at Monte Renoso (Corsica, France). **(c)** *L. pectinata* at Veleta peak (Granada, Spain). **(d)** *L. pulverulenta* at Puerto de Villatoro (Avila, Spain). **(e)** *L. pallida* subsp. *pallida* at Puerto de Villatoro (Avila, Spain). **(f)** *L. pallida* subsp. *virescens* at Sierra de Vicort (Zaragoza, Spain). **(g)** *L. cuneata* subsp. *valdes-bermejoi* at Sierra del Brezo (Palencia, Spain). **(h)** *L. flaveola* at Peña Trevinca (Orense, Spain). **(i)** *L. spathulifolia* at Sierra de Cazorla (Jaén, Spain), and **(j)** detail of the spathulate basal leaves. Photos by Salvatore Tomasello.

*Leucanthemopsis* (Giroux) Heywood is a small genus of the chamomile tribe of the daisy family (Compositae, Anthemideae). It is a polyploid complex including diploids, tetraploids, and hexaploids. According to the present taxonomy, the genus consists of ten species (Pedrol, 2019), eight of which are endemic to the Iberian Peninsula (Figure 1). The hexaploid, *L. longipectinata* (Font Quer) Heywood is a narrow endemic of the Rif Mountains of northern Morocco, whereas *L. alpina* (L.) Heywood is more widespread. The latter species is a polymorphic complex distributed in the south-western and central European alpine ranges (from the Pyrenees to the Carpathians) and comprises diploid, tetraploid, and hexaploid populations. Most of *Leucanthemopsis* species, especially the polyploid complexes (eight out of ten species), show a high degree of morphological polymorphism (Figure 2), and several infraspecific taxa have been described in the past (Heywood, 1975; Pedrol, 2019).

The onset of diversification processes in *Leucanthemopsis* has been estimated to be c. 4.4 million years (Ma) ago and the differentiation among the Iberian taxa was most likely within the last two million years (Tomasello et al., 2015). However, Tomasello et al. (2015) included only a few, mostly diploid samples, leaving phylogenetic relationships among Iberian taxa and temporal origin and diversification of polyploids unresolved.

In the present study, we therefore, aim at resolving the phylogenetic relationships within the western-Mediterranean genus *Leucanthemopsis*, including a comprehensive sampling (all species described and more individuals per species) and using sequence information from a number of single/low copy nuclear genes as well as plastid regions. More specifically, we investigate the mode (auto- vs. allopolyploid), the frequency, and the temporal framework of the formation of polyploids and thus contribute to improving our understanding of the importance of polyploid evolution for the biodiversity of the Mediterranean Basin.

## Materials and Methods

### Plant Material

Fifty-two accessions from members of the genus *Leucanthemopsis* were included in the present study. We used one to seven samples per taxon (17 taxa belonging to 10 *Leucanthemopsis* species). For population LPS168 of *L. cuneata* (Pau) Holub subsp. *cuneata*, two individuals were included, whereas from all other populations we used single individuals. Two additional accessions belonging to the sister genera *Prolongoa* Boiss. and *Hymenostemma* Kunze ex Willk. (Tomasello et al., 2015) were also included and used as outgroup in some of the analyses. Several accessions were collected by S. Tomasello in the summers of 2010 and 2011, silica-gel dried, and employed already in Tomasello et al. (2015), Tomasello and Oberprieler (2017), and Oberprieler et al. (2017). Additional samples were collected during the summers of 2015 and 2016 or sampled from herbarium specimens from the herbaria of the Real Jardín Botánico (MA), the Botanical Garden and Botanical Museum Berlin-Dahlem (B), and the Bavarian Natural History Collections Munich (M). This was done to include all taxa described by Heywood (1975) and Pedrol (2019), and to incorporate, when possible, material from *loci classici* or herbarium vouchers cited in the above-mentioned revisions. The importance of including topotypic material in phylogenetic studies involving intricate taxonomic groups has already been highlighted in other studies (Otero et al., 2021). A complete list of samples used in the present study is provided in the Supplementary Data Sheet 1. Herbarium vouchers for all the newly collected samples were deposited in the Herbarium Mediterraneum Panormitanum (PAL) and in some cases in the herbaria of the Real Jardín Botánico (MA), and of the University of Salamanca (SALA).

### Flow Cytometry

For samples collected in the field and dried in silica-gel, ploidy was estimated via flow cytometry mostly using five accessions per population. For populations collected in “Puerto de Paramera” (LPS139-LPS141), where the diploid *L. pulverulenta* (Lag.) Heywood grows sympatrically with the tetraploid, *L. pallida* subsp. *pallida* (Miller) Heywood, we measured all collected and surveyed individuals (ten individuals per population). We, therefore, estimated the ploidy level for 40 populations belonging to 11 *Leucanthemopsis* taxa. Approximately 10% of the samples were re-analyzed and used as replicates. *Petunia hybrida* E.Vilm. cv. PxPc6 was used as an internal standard (2C = 2.85 pg; Marie and Brown, 1993). Approximately 0.5–1 cm^2^ of leaf tissue of the standard and two- to three-fold tissue of the dehydrated sample material were employed. Nuclei were isolated by grinding the leaf material in Otto I buffer (Otto, 1992; Doležel and Göhde, 1995) and subsequently stained with 4′,6-diamidino-2-phenylindole (DAPI) in LB01 buffer (Doležel et al., 1989) modified with the supplement of ß-Mercaptoethanol (0.015 mM). Samples were measured on a PARTEC CyFlow^®^ Space instrument (Partec GmbH, Münster, Germany). For each sample, at least 8,500 nuclei were counted. The results were processed using the FloMax^®^ software (Partec GmbH, Münster, Germany). The ratio between the relative fluorescence of the sample nuclei and of those of the standard was used to estimate the ploidy of *Leucanthemopsis* accessions.

### Conceptual Framework for the Phylogenetic Analyses

We used a mixed approach including Sanger sequencing and (Roche-454) high throughput sequencing of amplicons to gather allelic information from single-copy nuclear markers and sequences from two plastid regions. Nuclear regions were carefully tested for recombinants/PCR artifacts and for paralogy using gene phylogenies and looking at highly supported clades emerging from long branches and including samples from different taxa.

**FIGURE 3:**
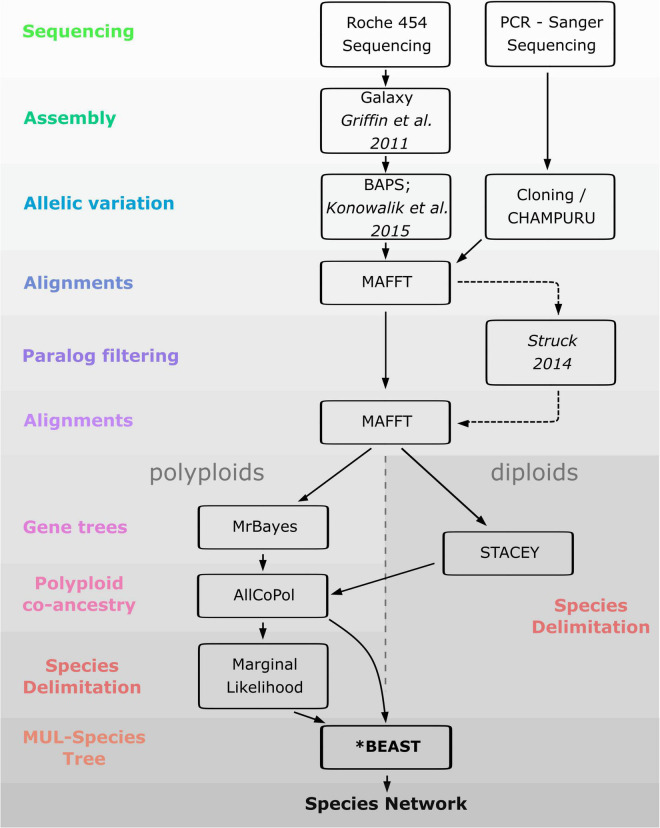
Workflow of the analyses performed in the present study.

As a prerequisite for the reconstruction of polyploid networks, coalescent-based species delimitation approaches were first used to circumscribe diploid lineages. We then investigated the mode of formation of polyploids (auto- vs. allopolyploids) by using allelic information along with methods capable to sort alleles in putative parental sub-genomes and infer species networks. Since taxon circumscription of polyploids of the Iberian Peninsula is controversial, we also estimated the number of times (allo)polyploidization took place independently by using marginal likelihood calculations for different taxon-circumscription hypotheses. Finally, we used coalescent-based methods to roughly estimate the age of the polyploid lineages and the temporal framework of reticulation events. The workflow of the analyses performed in the study is depicted in Figure 3.

### DNA Extraction, Amplification, and Library Preparation

Deoxyribonucleic acid extracts were obtained using either the DNeasy Plant Mini Kit (Qiagen, Venlo, Netherland) in the laboratory of the CSIC “Real Jardín Botánico” in Madrid or a modified protocol based on the CTAB method by Doyle and Doyle (1987) at the Institute of Plant Sciences of Regensburg University.

For downstream phylogenetic analyses, two plastid markers (the intergenic spacer regions *psbA-trnH* and *trnC-petN*) and five single-copy nuclear markers (*B12*, *B20*, *C12*, *C16*, and *D35*; Chapman et al., 2007) were employed. The plastid spacer region *psbA-trnH* was amplified using the primers *psb*Af and *trn*Hr (Sang et al., 1997), whereas for the *trnC-petN* spacer region, we used the primers *trn*C (Demesure et al., 1995) and *pet*N1r (Lee and Wen, 2004). PCR amplifications were performed using the *Taq* DNA Polymerase Master-mix Red (Ampliqon/Biomol, Odense, Denmark) in a final volume of 12.5 μl, and according to the protocol suggestions of the manufacturer. The following temperature profile was employed: 2′ at 95°C, then 36 cycles of 30′′ at 95°C, 60′′ at 62°C, and 60′′ at 72°C, with a final extension of 5′ at 72°C. PCR products were purified using Agencourt AMPure magnetic beads (Agencourt Bioscience Corporation, Beverly, MA, United States). Cycle sequencing was performed with the DTCS Sequencing Kit (Beckman Coulter, Fullerton, CA, United States), following the protocol suggested by the manufacturer. Sequences were analyzed on a CEQ 8000 sequencer (Beckman Coulter, Fullerton, CA, United States) and the obtained electropherograms were carefully checked for ambiguities using CHROMAS LITE v2.10^1^ (Technelysium Pty Ltd., Tewantin, Australia) and/or FINCHTV v1.3.1 (Geospiza Inc., Seattle, United States). When necessary, we used the IUPAC codes to indicate single nucleotide polymorphisms (SNPs). In the electropherograms, a site was considered polymorphic when more than one peak was present and the weakest one reached at least 25% of the intensity of the strongest one (Fuertes Aguilar et al., 1999; Mansion et al., 2005).

Allelic variation for four of the five single-copy nuclear markers (all except for *D35*) was assessed via Roche-454 high throughput pyrosequencing. Prior to sequencing, amplicons went through two rounds of PCRs. In the first round, the Peqlab KAPAHiFi polymerase (Peqlab Biotechnologie GmbH, Erlangen, Germany) was used to reduce PCR errors as much as possible. The forward primers used for the amplifications were those from Chapman et al. (2007), tailed with a 29 bp-long M13-forward primer. The GS FLX Titanium Primer B sequence was added to the reverse primers. The reverse *C12* primer was designed to obtain *Leucanthemopsis* amplicons shorter than 350 bp (Supplementary Table 1). The PCRs were performed in a final volume of 15 μl, following the manufacturer’s instructions and using the following “touch-down” program: 95°C for 5′; 20′′ at 98°C, 30′′ at 65–61°C, and 30′′ at 72°C for 5 cycles; finally 35 cycles of 98°C for 20′′, 60°C for 30′′, and 72°C for 30′′, with a final extension step of 72°C for 5′.

After purification of the PCR products, the second PCR round was performed to add sample-specific 4–5 bp long barcodes [i.e., Multiplex identifiers (MIDs)] to the amplicons. Therefore, the forward primers consisted of the following sequence combination: GS_FLX_Titanium_Primer_A–MID–M13-tail, while the reverse primer was always the GS_FLX_Titanium_Primer_B. A two-step PCR program was employed: 3′ at 95°C; 20 cycles of 20′′ at 95°C, and 1′ at 68°C; with a final extension of 5′ at 72°C. After purification of PCR products, concentrations and fragment lengths were measured, and libraries were pooled in a way that allowed for an appropriate sequence coverage to detect all alleles in diploids, tetraploids, and hexaploids (as described in Oberprieler et al., 2017). Sequencing was performed on a Roche-454 pyrosequencing machine at Microsynth (Balgach, Switzerland).

The fifth single-copy nuclear marker, *D35* included a simple sequence repeat (SSR) or microsatellite motive. Because of this, even diploid accessions often showed alleles that differed in length. This allowed us to decrypt the different alleles by using forward and reverse sequence information as described by Flot et al. (2006) and to avoid next-generation sequencing or cloning. The amplicons of this marker were Sanger-sequenced and alleles were detected in diploids with the software CHAMPURU v1.0 (Flot, 2007), whereas in tetraploids, they were deciphered manually. Since this method is not applicable when more than four sequences of different lengths overlap in the electropherograms, we cloned the hexaploid accessions [two *L. alpina* subsp. *pyrenaica* (Vierh.) Tomasello & Oberpr., two *L. cuneata* subsp. *cuneata*, and one *L. longipectinata*]. For this purpose, we used the CloneJET PCR cloning kit (Fermentas, Waltham, MA, United States) according to the manufacturer’s recommendations. Twenty-seven clones were picked for each accession, in order to have a probability of 0.95 to get sequence information for all possible alleles (refer to the formula proposed by Joly et al., 2006).

### Assembly, Allele Detection, and Alignment of Markers

For those samples and markers sequenced on a Roche-454 sequencing machine, reads were assembled using R (R Development Core Team, 2008) and the Galaxy web portal (Giardine et al., 2005; Goecks et al., 2010) as described by Griffin et al. (2011). Reads were assigned to marker regions using forward-primer sequences and to accessions using the sample-specific MIDs. Reads with Phred scores below 20 in more than 10% of the nucleotide positions were discarded. Potential alleles and/or chimerical sequences were identified using BAPS v5.2 (Corander et al., 2006, 2008; Cheng et al., 2011) and the procedure described by Konowalik et al. (2015). Accordingly, clusters with read numbers above the minimum expected for an allele, considering the ploidy of the sample and the total number of reads gained for the specific sample/marker combination (Oberprieler et al., 2017), were kept as possible allelic variants. By applying the “admixture based on mixture clustering” option in BAPS to the previously found clusters, reads of intermediate position, potentially resulting from alleged recombination during PCRs, were pinpointed and discarded after an additional visual inspection in ALIVIEW v1.18 (Larsson, 2014). Once alleles were identified, reads were collapsed into consensus sequences, applying a 20% threshold as a criterion to retain intra-allelic polymorphisms by scoring them as IUPAC-coded wobble nucleotide positions. Consensus sequences (alleles) were then marker-wise processed in ALIVIEW and aligned using MAFFT v6.833b (Katoh et al., 2002; Katoh and Toh, 2008).

Sequences from direct sequencing (e.g., from the plastid regions), cloning, or CHAMPURU (*D35*) were directly added to the alignments and aligned using MAFFT.

### Paralog Filtering

Although the selected nuclear regions are supposed to be single-copy in members of Compositae (Chapman et al., 2007), we checked for putative paralog sequences in the alignment following a similar approach to the one described in Struck (2014). Following this approach, clades with high support values are first identified as paralogy suspects. Secondly, those clades with long stems and comprising taxa with independent *a priori* evidence of monophyly together with other taxa are considered paralogs.

Therefore, we inferred neighbor-net networks for each of the different nuclear regions using SPLITSTREE v4.14.6 (Huson and Bryant, 2006). We used the General Time Reversible (GTR) model to estimate the genetic distances with estimated site frequencies and maximum likelihood (equal rates of site variation). Then, we excluded from the alignments all sequences of the resulting networks forming well-supported clades separated by long branches and comprising sequences from different taxa.

### Species Delimitation for the Diploid Taxa

Before proceeding with analyses including polyploid samples, we decided to apply coalescent-based species delimitation to the diploid accessions. We have done so because (i) diploid specimens need to be assigned to species (i.e., progenitor lineages) prior to allele co-ancestry analyses with polyploids (see “Gene Trees and Haplotype Network Inference”), (ii) we were not sure if diploid species or their numerous infraspecific taxa (Heywood, 1955, 1975) should be used as progenitor lineages, and (iii) species identification in some of the diploid taxa was considered problematic, especially concerning the diploid *Leucanthemopsis* populations growing in the central and eastern part of the Iberian Peninsula.

We used the BEAST2 (Bouckaert et al., 2019) package STACEY v1.2.1 (Jones, 2017a) to infer species boundaries among the diploid taxa described in *Leucanthemopsis*. STACEY uses a Bayesian approach to infer species delimitation and species phylogeny based on the multispecies coalescent model. It is one of the few model-based programs able to do so, demonstrating to outperform other methods while estimating the correct ultrametric species tree (Andermann et al., 2019) or inferring the best species delimitation scenario (Tomasello, 2018).

We used BEAUTI v2.6 (Bouckaert et al., 2019) to create an input file for STACEY. We included five nuclear loci and two plastid markers. Only diploid ingroup accessions were included in the analyses. During the inference, sequence substitution, clock- and gene-trees models were considered unlinked across loci. Sequence-substitution models were selected *a priori* in MODELTEST-NG (Flouri et al., 2015; Darriba et al., 2020) for each locus separately using the Bayesian Information Criterion (BIC). In the STACEY.xml input file, parameters of the substitution models were fixed to those found in MODELTEST-NG (Supplementary Table 2). The strict clock was enforced for all loci. The average clock rate of a random locus was fixed to one, while all other clock rates were estimated in relation to this locus. We set the “Collapse Height” to 1 × 10^–4^. This parameter has no biological meaning, and values between 0.000001 and 0.0001 usually produce similar results in similar runtimes (Jones, 2017a). The “Collapse Weight” parameter was estimated using a normal prior distribution with mean = 0.95 and σ = 1.0. We assigned to the bdcGrowthRate prior a lognormal distribution (*M* = 4.6, *S* = 1.5), a gamma shape (α = 0.1 and β = 3.0) to the popPriorScale prior, and a gamma prior (α = 1.0, β = 1.0) to the relativeDeathRate.

The input files were run for 1 × 10^8^ iterations, sampling every 1 × 10^4^th generation. Two independent runs were performed to check the convergence among independent analyses. Convergence and ESS (values > 200) were checked in TRACER v1.7 (Rambaut et al., 2018). Output files containing the trees sampled in the two independent runs were combined using LOGCOMBINER v2.6 (Bouckaert et al., 2019), after discarding 10% of the sampled iterations as burn-in. The obtained file was processed with the “SpeciesDelimitationAnalyser”^2^ (speciesDA.jar; Jones et al., 2015) using a “collapse height” of 1 × 10^–4^ and setting the similarity cut-off to 0.9. Finally, we produced a similarity matrix using a modified version of the R script provided by Jones et al. (2015).

### Gene Trees and Haplotype Network Inference

In *D35*, the alignment region between positions 249 and 294, characterized by a microsatellite motive (see “Species Delimitation For the Diploid Taxa”) producing non-informative homoplastic differences among sequences, was excluded from analyses. Indels in all marker alignments were coded as binary characters using the simple gap-coding method of Simmons and Ochoterena (2000) as implemented in the software program GAPCODER (Young and Healy, 2003). Bayesian inference (BI) phylogenetic analyses were performed in MRBAYES v.3.2.7 (Ronquist et al., 2012) for the plastid markers concatenated in a single alignment, and for each of the five nuclear regions separately. We used MODELTEST-NG and the Bayesian Information Criterion (BIC) to choose the best model of nucleotide substitution for each of the datasets (Supplementary Table 3). The analyses were run using two individual runs with three heated and one cold chain each, and a chain heating parameter of 0.2. The Metropolis-coupled Markov Chain Monte Carlo (MC^3^) chains were executed for 10,000,000 generations, sampling trees every 1,000th generation. To check convergence between individual runs, we considered the average standard deviation of split frequencies (acceptable when less than 0.01) and compared likelihood values and parameter estimates in TRACER v.1.7. Trees sampled during the Bayesian search were finally used as input to estimate allele co-ancestry in polyploids (see “Allele Co-ancestry in Polyploids”).

Additionally, plastid regions were used to reconstruct a haplotype network using the software POPART v.1.7^3^ and the TCS network algorithm (Clement et al., 2002). Samples, LPS135-10 (*Prolongoa hispanica*) and LPS180-1 (*L. alpina*) were excluded due to two long deletions in *trnC-petN* introducing ambiguities in the haplotype network reconstruction.

### Allele Co-ancestry in Polyploids

To assess allele co-ancestry in polyploid taxa, we used ALLCOPOL v.0.1.2 (Lautenschlager et al., 2020). This program uses allele permutations and infers the number of deep coalescences to assign alleles to diploid sub-genomes, as proposed by Oberprieler et al. (2017). ALLCOPOL uses heuristic approaches to overcome computational constraints encountered when increasing ploidy, number of genes, or samples.

Input for ALLCOPOL was produced by randomly choosing 500 gene trees from those sampled during the Bayesian search (excluding the first 1,000 trees) in each nuclear gene and in the concatenated plastid dataset. Gene trees (3,000 in total) were rooted using *Prolongoa*. During the heuristic search, we used the hill-climbing algorithm, with reinitialization (–u) and 1,000 iterations. We estimated allele co-ancestry for all polyploid taxa used in the different species-delimitation scenarios (see “Species Delimitation in Polyploids”). We ran the analyses twice for each taxon to check if the results were reproducible. In few cases, results were identical in terms of the number of extra lineages but slightly different concerning allele assignment. In these cases (which may indicate autopolyploidy), we calculated the probability of the gene trees under the two allele partition results using the CalcGTProb command (Yu et al., 2012) in PHYLONET (Than et al., 2008). We proceeded then with the allele co-ancestry receiving the best likelihood score.

### Species Delimitation in Polyploids

**FIGURE 4:**
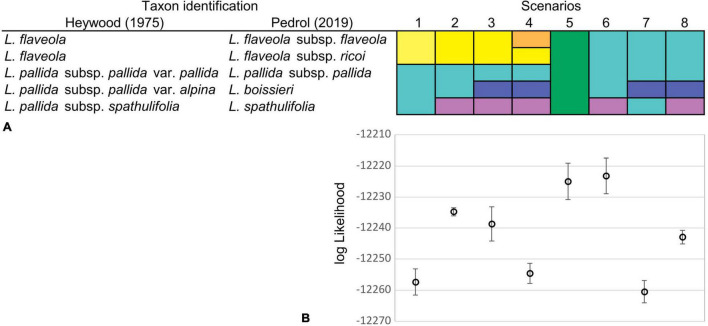
**(A)** Species delimitation scenarios were investigated for the Iberian tetraploid taxa of *Leucanthemopsis*. Taxa recognized by Heywood (1975) and Pedrol (2019) were merged into different potential tetraploid lineages. Different colors in a scenario denote different lineages delimited (e.g., in scenario one, two species are delimited). **(B)** Means and standard deviations of marginal-likelihood estimates from five replicate analyses for each of the species classification scenarios of the Iberian tetraploid taxa. Marginal likelihoods were estimated via Path Sampling (PS) in the Model Selection package v1.3.4 of BEAST2.

Since unequivocal taxon delimitation among tetraploid taxa of the Iberian Peninsula can be problematic in *Leucanthemopsis* and since we wanted to estimate the number of times (allo)polyploidization took place independently (how many reticulation events happened), we calculated marginal likelihoods for different delimitation scenarios of the Iberian tetraploid taxa via the Path Sampling (PS) method (Baele et al., 2012). The tested classification hypotheses followed Heywood (1975) and Pedrol (2019) both at species and subspecies levels, including classifications treating *L. pallida* subsp. *pallida* and *L. flaveola* (Hoffmanns. & Link) Heywood as a single species, and a model treating all tetraploids as a single specific evolutionary unit. In total, eight different species-delimitation hypotheses were investigated (Figure 4A). For each classification model, we performed five independent runs and calculated the means and standard deviations of marginal-likelihood values. The marginal likelihood was estimated from 60 path steps, each run lasting for 5,000,000 generations and using a pre-burn-in of 10,000 generations. For these analyses, we used the MODELSELECTION package v.1.3.4 in BEAST2 applying a 10% burn-in.

### Network Reconstruction and Age Estimation

We inferred a multilabel (MUL-)species tree assigning polyploid samples to taxa according to the species delimitation scenario having received the best marginal-likelihood score (see “Species Delimitation in Polyploids”). Subsequently, a species network was constructed by joining the leaves of the MUL-species tree (Lott et al., 2009; Oberprieler et al., 2017).

Input files for the BEAST analysis were prepared in BEAUTI v2.6 using the “*BEAST” template. We unlinked substitution models for all loci. Best substitution models were selected in MODELTEST-NG (see “Gene Trees and Haplotype Network Inference”; Supplementary Table 3). We incorporated into the analyses indel information, assigning to gap partitions a Mutation Death model. Clock and tree models of indel partitions were linked to those of the corresponding loci, while being kept unlinked across loci.

To be able to set the best clock model for each gene, we calculated marginal likelihoods via the PS method. The marginal likelihood was estimated from 100 path steps, each run for 1,000,000 generations with a pre-burn-in of 10,000 generations and a 10% burn-in. A difference of more than 3 log-likelihood units was considered as strong evidence for the acceptance of the relaxed (more parameter-rich) model against the strict clock (Kass and Raftery, 1995, Aydin et al., 2014). Since the relaxed clock was preferred in all cases (Supplementary Table 4), we used the “LogNormal Relaxed Clock” for all loci, giving to the ucldMean prior a lognormal distribution with an initial value of 0.00166 and 95% probability density ranging over two orders of magnitude (*M* = 0.013; *S* = 2.0). We have done so to give an informative prior to clock rates and assuming a standard substitution rate in plants of 5e^–9^ (Wolfe et al., 1989) and an average generation time of 3 years in *Leucanthemopsis* (considering that members of the genus are plurennial and not flowering during the first growing season). An exponential prior (mean = 1.0, offset = 0.0) was given to the ucldStdev.

For a better divergence time estimation, we decided to use a secondary calibration point besides the informative prior given to the clock rates (Tomasello et al., 2020). For this, we applied a normally distributed prior to the crown age of *Leucanthemopsis*, ranging between 2.9 and 5.9 Ma (mean = 4.39 Ma, σ = 0.91 Ma), which corresponds to the 95% highest posterior density (HPD) of the age estimated in Tomasello et al. (2015). We used the Calibrated Yule model as prior on the species tree. A broad uniform prior was applied to the birthRate (ranging from 0 to 1,000), whereas a 1/x prior (offset = 0.0) was given to the popMean. Two independent analyses were run for 4 × 10^8^ generations, sampling every 20,000th generation. Effective sample size (ESS) and convergence between independent analyses were checked in TRACER v1.7. Results of the two analyses were merged using LOGCOMBINER v2.6 applying a burn-in of 10%. Finally, the remaining 18,000 trees were used to construct a maximum clade credibility tree with a posterior probability limit set to 0.5 and “Mean Heights” for node heights using TREEANNOTATOR v2.6 (Bouckaert et al., 2019).

A species network was subsequently generated from the MUL-species tree by joining leaves belonging to sub-genomes of the same polyploid lineage. Branches with posterior probability lower than 0.7 were collapsed in the species network. Intervals of 95% HPD of the age of a polyploid taxon were obtained by merging the 95% HPD of the estimated divergence times of the sub-genomes involved in its formation.

## Results

### Genome Size and Ploidy Level

The quality of the flow-cytometric measurements in most of the cases was high, with average coefficient of variation (CV) values equal to 3.13% (±0.70) and 3.93% (±0.91) for the *Petunia* standard and the samples, respectively. Fluorescence ratios between replicate measurements varied on average by 0.02% (±0.06). Mean relative DNA content (fluorescent ratio between *Leucanthemopsis* and *Petunia* peaks) ranged from 3.27 to 3.86 in diploids, from 5.76 to 6.28 in tetraploids, and from 7.43 to 7.86 in hexaploids. In almost all cases, the ploidy of the measured samples corresponded to the expected one based on literature. Table 1 reports averaged taxon values for the relative DNA content and ploidy. Detailed information on all measures is available in Supplementary Data Sheet 2.

**TABLE 1:** Averaged fluorescent ratios (relative DNA content) and inferred ploidy for different taxa of *Leucanthemopsis* as obtained by flow-cytometric measurements.

Taxon	2*n*	*Leucanthemopsis*/*Petunia* ratio		Ploidy	Individuals	Populations
		Mean	SD			
*L. alpina* 2*x*	18	3.278	0.149	2*x*	21	4
*L. alpina* 4*x*	36	5.804	0.384	4*x*	10	2
*L. alpina* 6*x*	54	7.861	0.190	6*x*	10	2
*L. boissieri*	36	5.759	0.213	4*x*	10	2
*L. cuneata* subsp. *cuneata*	54	7.431	0.079	6*x*	5	1
*L. cuneata* subsp. *valdes-bermejoi*	18	3.420	0.106	2*x*	5	1
*L. flaveola* subsp. *flaveola*	36	5.847	0.072	4*x*	5	1
*L. flaveola* subsp. *ricoi*	36	5.781	0.120	4*x*	18	4
*L. heywoodii*	18*	3.307	0.066	2*x*	10	2
*L. pallida* subsp. *pallida*	36	6.227	0.265	4*x*	28	5
*L. pallida* subsp. v*irescens*	18	3.477	0.342	2*x*	7	3
*L. spathulifolia*	36^†^	6.118	0.173	4*x*	25	5
*L. pectinata*	18	3.722	0.215	2*x*	10	2
*L. pulverulenta*	18	3.866	0.143	2*x*	42	7

2n, chromosome numbers are those reported in the literature (refer to Supplementary Data Sheet 1); *Küpfer (1972) reported n = 9 for individuals ascribable to L. heywoodii (chromosome number expected for a diploid), whereas Pedrol (2019) considered the species being tetraploid; ^†^Antunez-Matarrubia (1981) reported a single count of 2n = 38 for L. spathulifolia, all other counts found in the literature were 2n = 36. Detailed information on flow-cytometric measurements is provided in Supplementary Data Sheet 2.

### Sequencing and Paralog Filtering

Library preparation and equimolar mixing worked well, and we did not have missing data for any sample, although the number of reads varied considerably across markers and accessions. In total 24,664 reads were obtained, and after quality trimming, about 83% of the reads were retained (20,429 reads; Supplementary Data Sheet 3). The number of alleles per individual was in most of the cases not higher than expected based on the ploidy level of the samples and under the assumption of each nuclear region being single-copy (Supplementary Data Sheet 3). After paralog filtering, 11 consensus sequences (alleles) were deleted from *B12*, 21 from *B20*, five from *C16*, and one from *D35*.

### Plastid Haplotype Network

**FIGURE 5:**
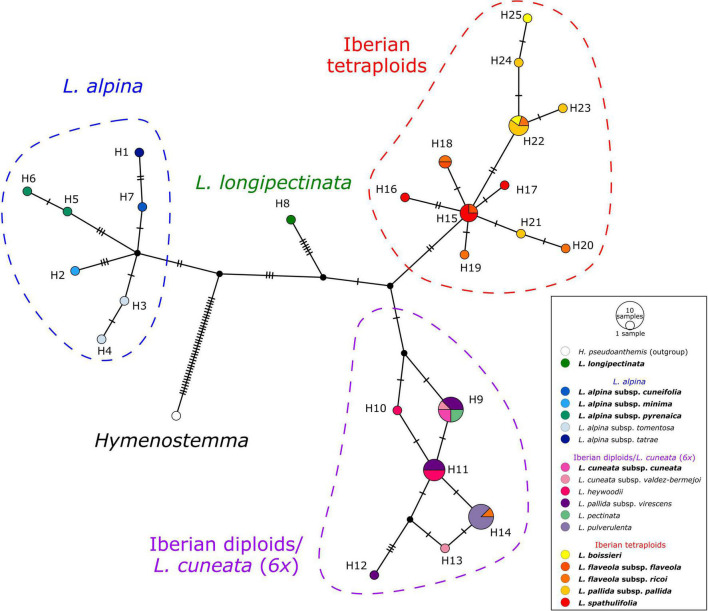
TCS haplotype network of *Leucanthemopsis* based on two plastid intergenic spacers, *psbA-trnH* and *trnC-petN*. Circle (haplotypes) sizes are proportional to the number of samples. Small black circles represent hypothetical haplotypes and tick lines are mutational steps separating haplotypes. Colored dashed lines indicate haplotype groups (blue: *L. alpina* s.l.; red: Iberian tetraploids; purple: Iberian diploids), whereas the colors of haplotypes correspond to the taxa as in the legend and Figure 1. Names of the taxa including polyploids are in bold.

The haplotype network obtained from the plastid spacers *psbA-trnH* and *trnC-petN* (Figure 5) showed four well-defined haplotype groups. The first group (blue in Figure 5) included accessions of *L. alpina*, the second (green) comprised the hexaploid Moroccan *L. longipectinata*. Further, the Iberian taxa were divided into two main haplotype groups, not corresponding to the present taxonomy, but rather to ploidy levels: one (red) was formed by only tetraploid accessions, whereas the other (purple) included mainly diploids. The hexaploid *L. cuneata* subsp. *cuneata* (from Sierra de Urbión, north-eastern Spain), had the same haplotype as the Iberian diploid *L. pectinata* (L.) G.López & C.E.Jarvis, some samples of *L. pallida* subsp. *virescens* (Pau) Heywood, and one of the two samples of *L. cuneata* subsp. *valdes-bermejoi* Pedrol. In the group containing only tetraploid taxa, haplotypes sampled in *L. spathulifolia* (J. Gay) Fern.Casas and a few samples of *L. flaveola* occupied a more proximal position in the network, whereas haplotypes found in *L. pallida* subsp. *pallida* and *L. boissieri* were more derived. Interestingly, one sample of *L. flaveola* had a haplotype otherwise found only in the diploid *L. pulverulenta* (H14 in Figure 5).

### Species Delimitation in Diploids

**FIGURE 6:**
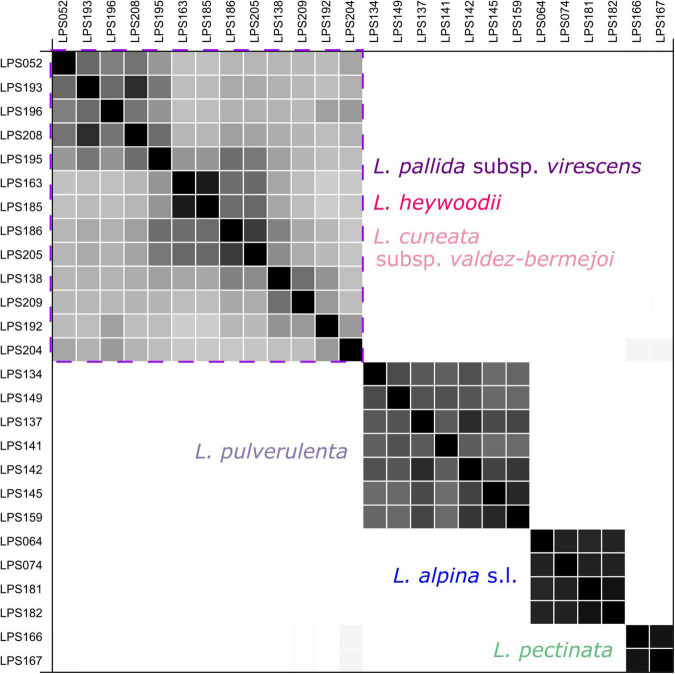
Similarity matrix produced by the coalescent-based species-delimitation analyses (STACEY) for the diploid members of *Leucanthemopsis*. Posterior probabilities for pairs of individuals belonging to the same cluster (species) are shown in black (1.0) and white (0.0). The purple square encloses the cluster including samples of *L. cuneata* subsp. *valdes-bermejoi, L. heywoodii*, and *L. pallida* subsp. *virescens*. The colors correspond to those of Figure 1.

“SpeciesDelimitationAnalyser” found a classification with four clusters (species) as the one with the highest frequency. The similarity matrix (Figure 6) also shows four distinct clusters, i.e., *L. pectinata*, *L. pulverulenta*, a cluster formed by the diploids of *L. alpina*, and a cluster including samples ascribable to *L. cuneata* subsp. *valdes-bermejoi*, *L. heywoodii* Pedrol, and *L. pallida* subsp. *virescens*. Individuals from one cluster had zero posterior probability of belonging to any other cluster.

### Species Delimitation in Polyploids

The species-delimitation scenario producing the best marginal-likelihood scores was scenario 6 (log-likelihood mean = −12,222.28; SD = 4.36; Figure 4B), in which tetraploid samples from the Iberian Peninsula were divided into two specific evolutionary units: (1) *L. spathulifolia* and (2) all other tetraploid samples considered being a single species (Figure 4A). Scenario 5, in which all tetraploid Iberian taxa were treated as a single species, was the second-best, receiving log-likelihood values partially overlapping with those of Scenario 6 (Supplementary Table 5).

### Species Network and Divergence Times

The MUL-species tree obtained from the *BEAST analyses showed a clear separation among three clades in *Leucanthemopsis*. The earliest diverging lineage was *L. longipectinata*, which is found to be the sister to a non-supported clade formed by all other taxa (Supplementary Figure 1). The other two supported lineages (clades) include members of *L. alpina* (posterior probability PP 0.89) and all Iberian taxa (PP 1.0), respectively. Relationships among taxa/sub-genomes within these two clades were only partially supported (Supplementary Figure 1).

**FIGURE 7:**
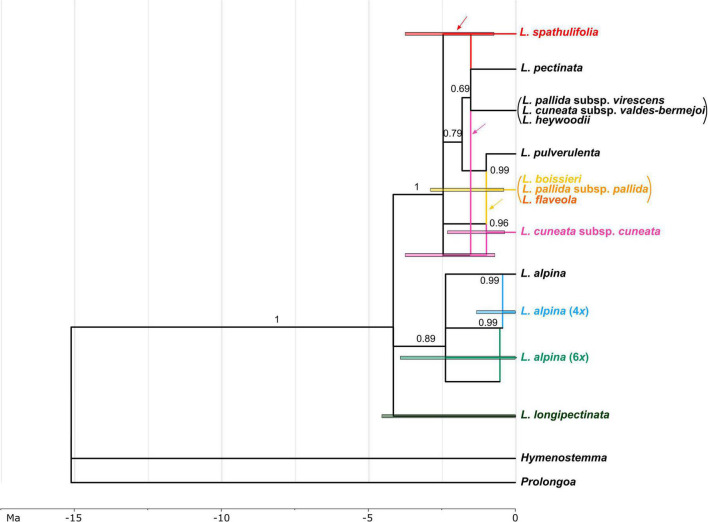
Network representing the phylogenetic relationships and reticulate evolution in *Leucanthemopsis*. Colors for polyploids correspond to those in Figure 1. Taxa merged into lineages by the species delimitation analyses are within parentheses (the diploids *L. cuneata* subsp. *valdes-bermejoi*, *L. heywoodii*, and *L. pallida* subsp. *virescens*, and the tetraploid *L. boissieri*, *L. flaveola*, and *L. pallida* subsp. *pallida*, respectively). The numbers above branches are posterior probabilities obtained in the *BEAST analysis. Branches that had posterior probability below 0.69 in the multi-labeled species tree (Supplementary Figure 1) were collapsed in the species network. Edges representing the maternal progenitor of a polyploid as inferred in ALLCOPOL are indicated by arrows. Colored bars indicate 95% highest posterior density (HPD) intervals of the age estimates. For polyploids, 95% HPD of age estimates were obtained merging the 95% HPD of the divergence times of the sub-genomes contributing to the polyploidy formation (see “Materials and Methods” for more details). The time scale is expressed in millions of years (Ma).

The Moroccan hexaploid, *L. longipectinata* was inferred as being autopolyploid, with differentiations among the three sub-genomes having occurred presumably between 4.54 and 0.2 Ma ago. Additionally, tetra- and hexaploid populations of *L. alpina* were reconstructed as autopolyploid, whereas the Iberian hexaploid, *L. cuneata* subsp. *cuneata* was found to be formed by the contributions of (1) an early-branching lineage of the Iberian clade, (2) a lineage related to *L. pectinata* and the other diploids growing along the eastern part of the Iberian Peninsula (i.e., *L. cuneata* subsp. *valdes-bermejoi, L. heywoodii* and *L. pallida* subsp. *virescens*; Figure 7), and (iii) a lineage involved also in the formation of the Iberian tetraploids (i.e., *L. boissieri, L. flaveola*, and *L. pallida* subsp. *pallida*). In the origin of the Iberian tetraploids (including all the tetraploid taxa present in the Iberian Peninsula with the exclusion of *L. spathulifolia*) also the diploid *L. pulverulenta* took part. Finally, *L. spathulifolia* was formed by the contribution of a lineage related to *L. pectinata* and the complex including *L. cuneata* subsp. *valdes-bermejoi, L. heywoodii*, and *L. pallida* subsp. *virescens*, and (as in *L. cuneata* subsp. *cuneata*) an early-branching lineage of the Iberian clade. The formation of the Iberian polyploids was inferred to be the period between 2.87 and 0.59 Ma.

## Discussion

In the present study, we aimed at reconstructing the evolutionary history of the western Mediterranean genus *Leucanthemopsis* using comprehensive sampling and sequence information from single-copy nuclear genes and plastid markers. Both auto- and allopolyploidy were involved in the formation of polyploid taxa in the genus. In the Iberian Peninsula, allopolyploids originated during the early Pleistocene, probably thanks to latitudinal and elevational range shifts that brought isolated diploid *Leucanthemopsis* lineages into contact. Our study highlights the importance of the Pleistocene climatic oscillations and polyploidizations for the high plant diversity in the western Mediterranean Basin.

Flow-cytometric measurements were in line with previously reported ploidy levels based on chromosome counts except for *L. heywoodii*. We included four samples ascribable to this taxon (LPS192, LPS193, LPS208, and LPS209). Unfortunately, none of them was from the *locus classicus* (Sierra de Javalambre). However, all were from the distribution range of the species given by Pedrol (2019; i.e., Sierras de Gúdar, Albarracìn, Valdemeca, Javalambre). We determined the ploidy level of two populations (LPS208 and LPS209), which were found to be diploid. These two populations were visited and sampled by Juan Pedrol (among other botanists) in the summer of 2016. Additionally, population LPS209 (SALA158943) was identified by its collectors as *L. pulverulenta* subsp. *pseudopulverulenta* (Heywood) Heywood, a taxon that was subsequently synonymized with *L. heywoodii* by Pedrol (2019). We do not know on which data the tetraploid report provided by Pedrol (2019) is based. To the best of our knowledge, the only chromosome count ever published from a *Leucanthemopsis* accession from the *locus classicus* of *L. heywoodii* was diploid (Küpfer, 1972). Therefore, we included the samples ascribable to this taxon in the species delimitation analyses of the diploid dataset.

### Phylogenetic Relationships Among Diploid Members of *Leucanthemopsis*

The results from the STACEY analyses revealed four main genetic clusters (lineages), corresponding to *L. alpina, L. pectinata*, *L. pulverulenta*, and a cluster formed by *L. cuneata* subsp. *valdes-bermejoi*, *L. heywoodii*, and *L. pallida* subsp. *virescens* (Figure 6). The analyses did not support any differentiation within diploid *L. alpina*. *L. alpina* subsp. *tomentosa* (Loisel.) Heywood (LPS181 and LPS181 in the present study), endemic to the highest mountain peaks of Corsica, has been treated sometimes as an independent species (Loiseleur-Deslongchamps, 1807; De Candolle, 1837; Holub, 1977; Pignatti, 1979, 1982) due to its peculiar morphology and the isolated distribution range. In accordance with our previous study focused on this species complex (Tomasello and Oberprieler, 2017), however, our present results do not support such taxonomic treatment.

Within the cluster formed by *L. cuneata* subsp. *valdes-bermejoi, L. heywoodii*, and *L. pallida* subsp. *virescens*, a certain level of genetic structuring is visible, but without clear boundaries among the three taxa. The accessions of *L. cuneata* subsp. *valdes-bermejoi* (LPS163 and LPS185) received a high posterior probability of belonging together in the same species, but also show relatively high similarity with other samples of the cluster, especially with the *L. pallida* subsp. *virescens* populations collected in Pico Revolcadores (Murcia; i.e., LPS186 and LPS205). Otherwise, it is not possible to couple the observed phylogenetic patterns within the cluster with neither any morphological (e.g., white or yellow limb of ray florets) or ecological (growing on the siliceous or calcareous substrate) features nor with formerly recognized taxonomic units. As a consequence, this lineage is very heterogeneous as circumscribed in our STACEY analyses, and includes populations with yellow or white ray florets (i.e., the varieties *virescens* and *bilbilitanum* recognized by Heywood (1975) within *L. pallida* subsp. *virescens*), those growing in subalpine vegetation (*L. heywoodii*) as well as such from much lower altitudes (down to 800 m), mostly found on limestone, but in some cases also on siliceous outcrops [e.g., *L. pallida* subsp. *virescens* var. *bilbilitanum* sensu Heywood (1975)]. There have been attempts to acknowledge a such-circumscribed complex as a separate species (Valdés-Bermejo in Antunez-Matarrubia, 1981; Pérez-Romero et al., 2005). However, those attempts were not generally accepted later-on. Therefore, this complex surely deserves additional studies with a denser sampling and more powerful genetic markers.

In the MUL-species tree (Supplementary Figure 1) and in the species network (Figure 7), diploids of *L. alpina* are clearly separated from the Iberian diploids. In the clade constituted by Iberian diploids, *L. pulverulenta* is sister to the clade formed by *L. pectinata* and the lineages including all other Iberian diploids (see above). *L. pulverulenta* is a well-defined species, distinct from the other Iberian diploids in both morphological and ecological respects. In fact, this species has smaller leaves, both the basal and the stem ones being deeply pinnatisect, and ray florets always with white limbs. This species is a mesomediterranean element typical of sandy soils of the north-western and central Iberian *meseta* (Ladero and Velasco, 1978), whereas the other taxa are always, although to different extents, linked to mountain environments.

### Origin of Polyploids

We used ALLCOPOL (Lautenschlager et al., 2020) to sort alleles to putative parental sub-genomes and infer the origin(s) of polyploids. This allowed us to calculate marginal likelihoods for different taxon assignments and estimate the number of independent polyploidization events. Šlenker et al. (2021) resolved the origin of the allotetraploid *Cardamine barbaraeoides* Halácsy using target enrichment data. Lautenschlager et al. (2020) and Šlenker et al. (2021) sorted alleles of the tetraploids into parental sub-genomes. To do so, the latter used the position in the nuclear gene trees occupied by the homeologs of polyploids relative to the maternal progenitor. The authors demonstrated the scalability of their method to genomic data and provided the script “AlleleSorting.”^4^ However, the efficiency of the method needs to be proven (1) when information on the maternal progenitor is missing and (2) for ploidies higher than the tetraploid level. ALLCOPOL does not need the maternal progenitor of a polyploid to be sampled. Moreover, our relatively small dataset (up to the hexaploid level; a maximum 20 samples per polyploid lineage and no more than ten genomic regions) allowed the program to perform the analyses in a reasonable time.

The marginal-likelihood analyses supported the scenario with two independent origins of the Iberian tetraploids, i.e., one leading to *L. spathulifolia* and the other to all other Iberian tetraploids (Scenario 6 in Figure 4A). Also other pieces of evidence support a separate origin of *L. spathulifolia*, which possesses the least derived haplotypes in the group of the Iberian tetraploids (Figure 5). This taxon has a characteristic karyotype, with a pair of heteromorphic chromosomes, one of which is always smaller than the other (Fernandez-Casas, 1977; Antunez-Matarrubia, 1981), its leaf morphology is very peculiar, and pollen grains are smaller than in the other Iberian tetraploids (Antunez-Matarrubia, 1981). In addition, it grows strictly on limestone bedrock, whereas all other tetraploids are calcifuge (Heywood, 1975; Pedrol, 2019).

Concerning the origin of the Iberian tetraploids, the existence of an extinct diploid that contributed as a maternal progenitor in both tetraploid formations needs to be postulated. In fact, all Iberian tetraploids have a peculiar plastid type, well-distinct from those observed in other members of the genus (Figure 5). The same pattern is observable in the network reconstruction (Figure 7), although resolution is relatively low within this clade. This putatively extinct diploid must have diverged early from the other diploids in the Iberian clade. *L. spathulifola* was formed (apart from the above-mentioned extinct diploid) with the contribution of an ancestor of *L. pectinata* and the lineage including *L. cuneata* subsp. *valdes-bermejoi*, *L. heywoodii*, and *L. pallida* subsp. *virescens.* The affinity between *L. spathulifolia* and *L. pallida* subsp. *virescens* was highlighted by former botanists and even a possible autopolyploid origin of *L. spathulifolia* from the latter taxon has been hypothesized (Antunez-Matarrubia, 1981). The contribution of an *L. pallida* subsp. *virescens*-like progenitor would explain the morphological (this taxon bears slightly spathulate leaves) and ecological (both calcicole) affinity. Moreover, both taxa are mainly distributed in the eastern Iberian Peninsula, *L. pallida* subsp. *virescens* along the Iberian System and *L. spathulifolia* in the Baetic System. *L. spathulifolia* might have been formed during the past north-south species range shifts, when the diploid maternal progenitor came in contact with an *L. pallida* subsp. *virescens*-like progenitor in the Baetic System. Nevertheless, isolated populations ascribable to the lineage including *L. cuneata* subsp. *valdes-bermejoi, L. heywoodii*, and *L. pallida* subsp. *virescens* are still found not very distant from the species range of *L. spathulifolia* (e.g., LPS186 and LPS205 from Pico Revolcadores, Murcia).

The other tetraploidization event gave rise to *L. boissieri, L. flaveola*, and *L. pallida* subsp. *pallida*. *L. boissieri* was considered to be a variety of *L. pallida* subsp. *pallida* [i.e., *L. pallida* subsp. *pallida* var. *alpina* (Boiss. & Reuter) Heywood] until the most recent taxonomic treatment by Pedrol (2019). It differs from the typical variety by its white ray florets and the somewhat smaller overall habitus. It substitutes the typical variety above 2000 m in the highest peaks of the Central System. *L. flaveola* is considered to be the vicariant of *L. pallida* subsp. *pallida* toward the westernmost part of the Central System, in the mountain ranges between northern Portugal and the highest peaks between Galicia and Leon. The two taxa were often associated with each other and *L. flaveola* has sometimes been considered to be a subspecies of *L. pallida* (Ladero and Velasco, 1978). According to our analyses, *L. pulverulenta* was the other diploid progenitor, which is in line with the suggestion of Mariz (1891) that *L. pulverulenta* is a possible progenitor of *L. flaveola* and *L. pallida* subsp. *pallida*. Despite their different ecology, contact zones between *L. pulverulenta* and *L. pallida* subsp. *pallida* and/or *L. flaveola* are still observable along the Central Systems and, albeit only sporadically, hybridization might be ongoing to the present day (e.g., the *L. flaveola* accession LPS161 shares the haplotype with *L. pulverulenta*; Figure 5).

The hexaploid *L. cuneata* subsp. *cuneata*, a narrow endemic of the “Sierra de Urbión” in northern Spain, is reconstructed here as an allohexaploid (Figure 7). In this case, the maternal progenitor must have been from the lineage including *L. cuneata* subsp. *valdes-bermejoi*, *L. heywoodii*, and *L. pallida* subsp. *virescens*. It might be *L. cuneata* subsp. *valdes-bermejoi*, but this is difficult to confirm based on the haplotype network (Figure 5). It is likely that the maternal progenitor first hybridized with the extinct progenitor of the Iberian tetraploids, and then again with *L. pallida* subsp. *pallida* or *L. flaveola*. This hypothesis and the contribution of the former taxon as one of the progenitors of *L. cuneata* subsp. *cuneata* may receive some support from the classification proposed by Pau (1906), who first described this taxon as a variety of *L. pallida*.

The hexaploid, *L. longipectinata* clearly has an autopolyploid origin. It is the only representative of the genus in North Africa. According to the present temporal reconstructions, it diverged from the rest of the genus about 4 Ma ago. It is probably a descendant of a Tertiary *Leucanthemopsis* diploid confined to the North African mountain ranges, which gave rise to the actual hexaploid as a single extant taxon of this lineage that is limited to the high elevations of the northern Moroccan Rif Mountains. Alternatively, an allopolyploid origin within the African lineage followed by the extinction of all progenitors is also conceivable.

Additionally, polyploid members of *L. alpina* are of autopolyploid origin, too. This species is a polymorphic complex including diploid, tetraploid, and hexaploid populations. Whereas diploids are scattered in the western Alps, Corsica, the Dolomites, the western and the southern Carpathians, and the Dinaric Alps, the tetraploids are widespread across the distribution range of the whole species and the hexaploids are limited to the Central Pyrenees (Tomasello and Oberprieler, 2017; Tomasello and Konowalik, 2020). As amply elucidated by Tomasello and Oberprieler (2017), the polyploid members have been formed recursively and independently in different places of the species range.

### Spatio-Temporal Framework of Polyploidization Events in *Leucanthemopsis*

McCann et al. (2018) estimated the age of formation of allopolyploid taxa of *Melampodium* L. by using BEAST2 and treating sub-genomes of allopolyploids as “species.” These were forced to be monophyletic with the diploid progenitors and splits were forced to be contemporaneous using the cross-bracing strategy (Shih and Matzke, 2013). In contrast to that study, we had no *a priori* knowledge of the diploid progenitors of the polyploids. Moreover, one of the diploid progenitors is missing in our dataset (the presumably extinct progenitor of all Iberian tetraploids). As a consequence, we have estimated the age of polyploid formation by joining (*a posteriori*) the 95% HPD of the splits contributing to the different sub-genomes of an allopolyploid. This results in broader and rougher age estimates of polyploid formations.

Based on our results, the formation of polyploids, particularly those in the Iberian clade, took place in the early Pleistocene (between 2.87 and 0.59 Ma; Figure 7; Supplementary Figure 1). The transition between Pliocene and Pleistocene is well known for the abrupt climate change causing the onset of the major northern hemisphere glaciations at approximately 2.7 Ma (Lisiecki and Raymo, 2005). Also in the Iberian Peninsula, a cyclic climate regime with warm and humid interglacials and cold and dry glacials established during the early Pleistocene (Altolaguirre et al., 2019). The onset of glacial–interglacial oscillations might have produced latitudinal and elevational species-range shifts that brought into contact the once isolated *Leucanthemopsis* lineages. Accordingly, *L. spathulifolia* could have originated during one of the southward shifts of a *L. virescens*-like lineage that met the maternal ancestor of the Iberian tetraploids in the Baetic System. The mountain ranges of eastern Spain (Baetic System, the “Serrania de Cuenca,” the Iberian System until Urbión, and the Pyrenees) are well-known to have favored north-south migrational shifts of Oro-Mediterranean plant species (Rivas Martínez, 1969, 1973; Vargas, 2003).

The polyploidization event giving rise to the other Iberian tetraploids could have taken place between the maternal ancestor of all Iberian tetraploids and *L. pulverulenta*, presumably in the central and north-western Iberian Peninsula, where the latter taxon and most of the Iberian tetraploids still co-occur. This allo-polyploidization event must have been more recent, as evidenced by the younger divergence time (1.61–0.38 Ma ago) between *L. pulverulenta* and the sub-genome of the tetraploid derivatives (the second sub-genome in Supplementary Figure 1), placing the formation of this polyploid around the “Mid Pleistocene Transition” (∼0.8 Ma ago). If we assume that the maternal progenitor of the Iberian tetraploid was a montane or Oro-Mediterranean species, this could have met *L. pulverulenta* due to downward migrations during cold periods. The Mid-Pleistocene Transition is the time when high-amplitude of 100 ka glaciation cycles settled (Lisiecki and Raymo, 2005). In that period, glaciations became longer and more severe and alternation of cold and warm periods produced major latitudinal and elevational shifts in species-distribution ranges. Those shifts (and the resulting repeated isolation and collision events of diverging lineages) are considered one of the major forces driving plant speciation in southern Europe Quaternary (Tomasello et al., 2020; Kadereit and Abbott, 2021).

## Data Availability Statement

Sequences and raw reads presented for the first time in this study can be found under the ENA project PRJEB50763. For GenBank accession numbers of sequences already used in previous studies see Supplementary Material.

## Author Contributions

ST and CO conceived and designed the study and wrote the manuscript. ST collected plant material and performed the laboratory work. All authors contributed to the article and approved the submitted version.

## Conflict of Interest

The authors declare that the research was conducted in the absence of any commercial or financial relationships that could be construed as a potential conflict of interest.

## Publisher’s Note

All claims expressed in this article are solely those of the authors and do not necessarily represent those of their affiliated organizations, or those of the publisher, the editors and the reviewers. Any product that may be evaluated in this article, or claim that may be made by its manufacturer, is not guaranteed or endorsed by the publisher.

## Footnotes

1
http://technelysium.com.au/chromas.html

2
https://indriid.com/software.html

3
http://popart.otago.ac.nz/index.shtml

4
http://github.com/MarekSlenker/AlleleSorting
