# Supplementary material for: Reticulate Evolution in the Western Mediterranean Mountain Ranges: The Case of the Leucanthemopsis Polyploid Complex
Source: Front Plant Sci. 2022 Jun 17;13:842842. doi: 10.3389/fpls.2022.842842 (PMC9247603; doi:10.3389/fpls.2022.842842)
Supplement: Supplementary file 5 [file Data_Sheet_5.PDF]

frontiers

Supplementary Material

Supplementary Table 1. List of primers employed to amplify the chloroplast spacer and the nuclear single-copy genes, along with sequences of universal primers and oligonucleotides needed for the Roche 454 sequencing.

Region	Forward primer	Revers primer
psbA-trnH	GTTATGCATGAACGTAATGCTC	CGCGCATGGTGGATTCACAAATC
trnC-petN	CCAGTTCAAATCTGGGTGTC	CCCAAGCAAGACTTACTATATCC
B12	CAAGTGGCTGCAGCCATGGG	ACATCRGGMACCATTCCWCCGGTGT
B20	AGTGGWATYAGTGGKGCTAGTTACT	CCACCACGHACAAGMAGCCAAAG
C12	TCTTGCACCACCAACTGYTTGGC	GGGACAATGTTCAATGCTG
C16	ACAAGGCTTTTGGAATTGYCC	TTKCCAGCRAAATCATTWTCAGGRGT
D35	AAGGAGGMTTCATGGADTTYGACAA	CCWGTTTTGTCTGCTCTGAATTC
M13	CACGACGTTGTAAAACGAC	
TitA	CGTATCGCCTCCCTCGCGCCATCAG	
TitB	CTATGCGCCTTGCCAGCCCGCTCAG	

Supplementary Table 2. Alignment length, OTUs, model of nucleotide substitution selected by ModelTest-NG with the Bayesian Information Criterion (BIC), number of variable sites and parsimony informative sites for all regions used for the analyses including only diploids.

Region	OTUs	Length	Model	Γ	I	Variable sites	Parsimony informative
psbA-trnH	26	421	TPM3uf	-	-	8	5
trnC-petN	26	601	F81+I	-	0.937	12	8
B12	48	363	HKY+I	-	0.788	41	26
B20	33	322	TPM1	-	-	18	9
C12	56	309	HKY+ Γ	0.125	-	58	45
C16	38	201	HKY	-	-	22	15
D35	35	345	F81	-	-	13	6

Supplementary Table 3. Alignment length, OTUs, model of nucleotide substitution selected by ModelTest-NG with the Bayesian Information Criterion (BIC), number of variable sites and parsimony informative sites for all regions used for the analyses including all accessions (diploids and polyploids).

Region	OTUs	Length	Model	Γ	I	Variable sites	Parsimony informative
psbA-trnH	55	458	TPM3uf	-	-	35	14
trnC-petN	55	610	TPM1uf	-	-	37	13
B12	194	367	TPM3uf+ Γ	0.477	-	79	32
B20	99	325	TrNef+ Γ+I	0.523	0.756	50	23
C12	156	316	TPM1+Γ+I	0.546	0.359	89	56
C16	119	202	HKY	-	-	48	19
D35	127	349	HKY + Γ	0.342	-	20	10

Supplementary Table 4. Results from the clock model selection performed using Marginal Likelihood with the Path Sampling (PS) method in the Model Selection package v.1.3.4 of BEAST2.

**Clock**	***B12***	***B20***	***C12***	***C16***	***D35***	***psbA-trnH***	***trnC-petN1***
**Strict**	-2698.0619	-1327.9019	-3358.0892	-721.63365	-1567.8782	-1130.508	-1345.8106
**Relaxed**	-2689.3115	-1323.2857	-3353.0683	-705.63296	-1559.5526	-1122.2828	-1335.4751
Δ	-8.75	-4.616	-5.021	-16.001	-8.325	-8.225	-10.335

Supplementary Table 5. Marginal Likelihood calculation for the eight species classification scenarios tried for the Iberian tetrapoid samples. Marginal Likelihood was calculated using the Path Sampling (PS) method in the Model Selection package v.1.3.4 of BEAST2. We performed 5 independent calculations per scenario, and calculated mean and standard deviation.

	Scenario1	Scenario2	Scenario3	Scenario4	Scenario5	Scenario6	Scenario7	Scenario8
**Run1**	-12259.70942	-12236.18808	-12231.99558	-12255.53865	-12221.85318	-12224.1635	-12259.06769	-12240.5456
**Run2**	-12250.33693	-12233.49928	-12233.65665	-12253.16039	-12230.57609	-12225.51189	-12256.151	-12242.26214
**Run3**	-12259.80847	-12233.89111	-12244.38671	-12252.67311	-12225.88049	-12216.65136	-12259.29558	-12241.40247
**Run4**	-12256.54828	-12236.16161	-12240.23873	-12251.76149	-12222.60157	-12218.66236	-12265.44215	-12245.23691
**Run5**	-12260.53047	-12234.00979	-12243.04472	-12259.89972	-12223.91918	-12226.41587	-12262.64154	-12245.27524
**mean**	**-12257.38671**	**-12234.74997**	**-12238.66448**	**-12254.60667**	**-12224.9661**	**-12222.28099**	**-12260.51959**	**-12242.94447**
**SD**	**4.229225078**	**1.314403397**	**5.566895537**	**3.271482769**	**3.488749814**	**4.355082396**	**3.585560231**	**2.195772902**

Supplementary Figures

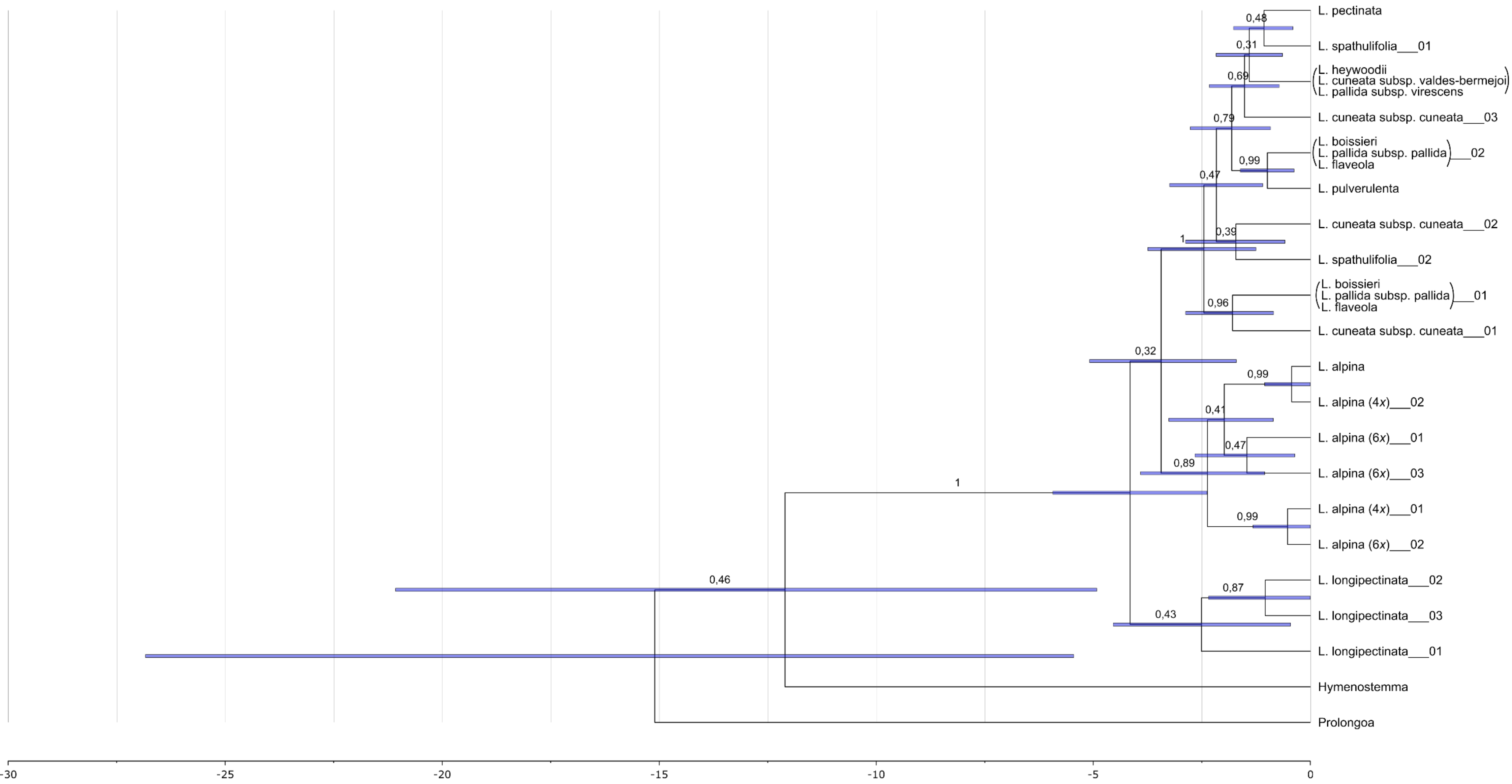

Supplementary Figure 1. MUL-species tree obtained from the *BEAST analyses and assigning samples to species according to the species delimitation Scenario 6. Polyploid lineages are represented in the tree by n leaves corresponding each to the parental sub-genomes contributing to the formation of the polyploid, as inferred in AllCoPol. Taxa merged into lineages inferred in the species delimitation analyses are written into parentheses (the diploids *L. cuneata* subsp. *valdes-bermejoi*, *L. heywoodii* and *L. pallida* subsp. *virescens*, and the tetraploid *L. boissieri*, *L. flaveola* and *L. pallida* subsp. *pallida*, respectively). Numbers above branches are posterior probabilities. Bars indicate 95% highest posterior density (HPD) intervals of the age estimate. Times scale is expressed in million years (Ma).

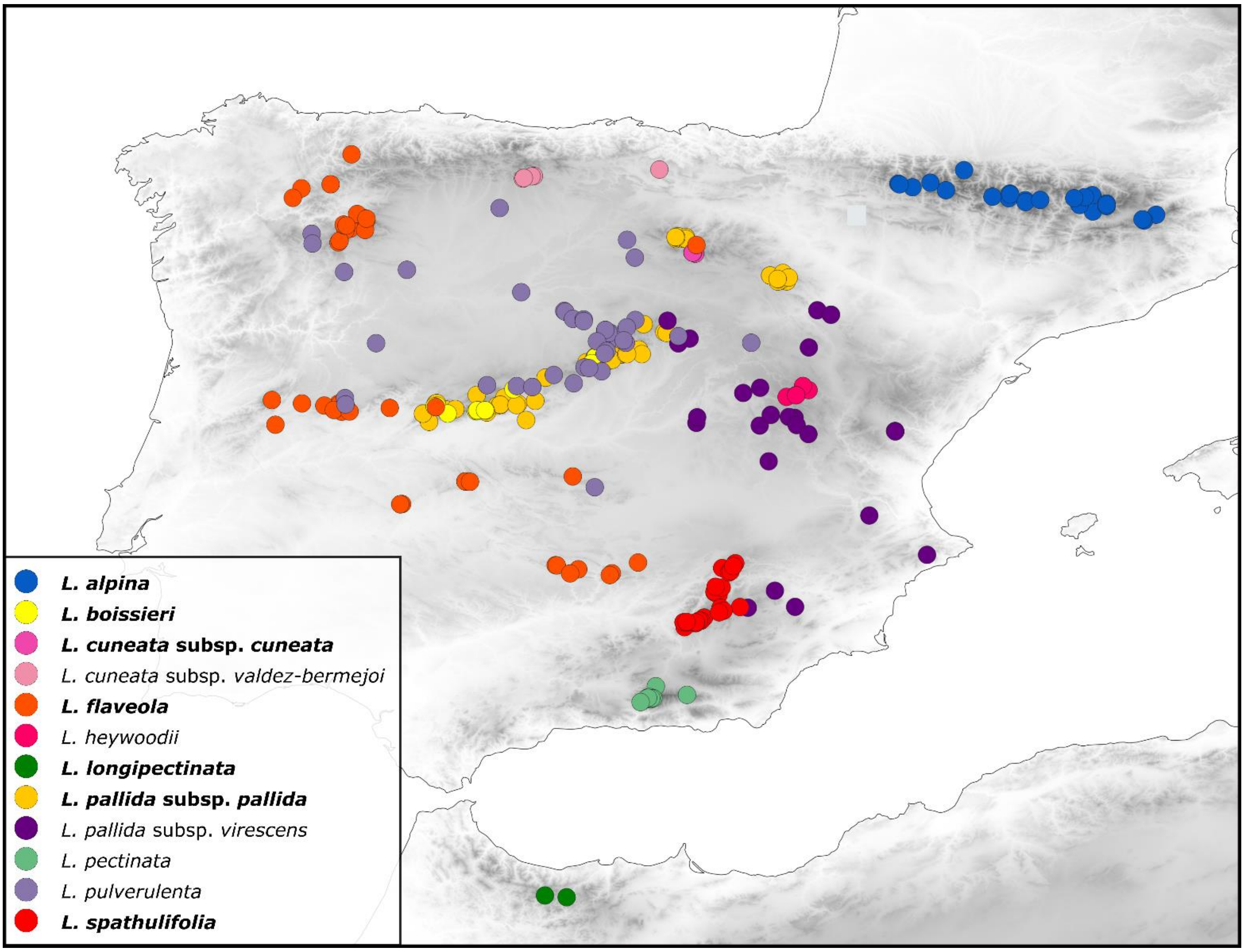

Supplementary Figure 2. Map displaying the georeferenced herbarium vouchers from Real Jardín Botánico (MA), the Botanical Garden and Botanical Museum Berlin-Dahlem (B) and the Bavarian Natural History Collections Munich (M) used to build the Map in Figure 1. Detailed information on the georeferenced herbarium vouchers is provided in Supplementary Data Sheet 4. In the legend, taxa including polyploids are in bold, whereas colours correspond to those in Figure 1.
